# Extracting secondary data from citizen science images reveals host flower preferences of the Mexican grass‐carrying wasp *Isodontia mexicana* in its native and introduced ranges

**DOI:** 10.1002/ece3.11537

**Published:** 2024-06-14

**Authors:** Nadja Pernat, Daniyar Memedemin, Tom August, Cristina Preda, Lien Reyserhove, Jens Schirmel, Quentin Groom

**Affiliations:** ^1^ Institute of Landscape Ecology University of Münster Münster Germany; ^2^ Centre for Integrative Biodiversity Research and Applied Ecology University of Münster Münster Germany; ^3^ Faculty of Natural and Agricultural Sciences Ovidius University of Constanta Constanţa Romania; ^4^ UK Centre of Ecology and Hydrology Wallingford UK; ^5^ Instituut voor Natuur‐ en Bosonderzoek, Team Oscibio Brussel Belgium; ^6^ RPTU Kaiserslautern‐Landau, iES Landau Institute for Environmental Sciences Landau Germany; ^7^ Meise Botanic Garden Meise Belgium

**Keywords:** biodiversity, citizen science, ecological interactions, invasion biology, pollinator, secondary data

## Abstract

We investigated the plant‐pollinator interactions of the Mexican grass‐carrying wasp *Isodontia mexicana—*native to North America and introduced in Europe in the 1960s*—*through the use of secondary data from citizen science observations. We applied a novel data exchange workflow from two global citizen science platforms, iNaturalist and Pl@ntNet. Images from iNaturalist of the wasp were used to query the Pl@ntNet application to identify possible plant species present in the pictures. Simultaneously, botanists manually identified the plants at family, genus and species levels and additionally documented flower color and biotic interactions. The goals were to calibrate Pl@ntNet's accuracy in relation to this workflow, update the list of plant species that *I. mexicana* visits as well as its flower color preferences in its native and introduced ranges. In addition, we investigated the types and corresponding frequencies of other biotic interactions incidentally captured on the citizen scientists' images. Although the list of known host plants could be expanded, identifying the flora from images that predominantly show an insect proved difficult for both experts and the Pl@ntNet app. The workflow performs with a 75% probability of correct identification of the plant at the species level from a score of 0.8, and with over 90% chance of correct family and genus identification from a score of 0.5. Although the number of images above these scores may be limited due to the flower parts present on the pictures, our approach can help to get an overview into species interactions and generate more specific research questions. It could be used as a triaging method to select images for further investigation. Additionally, the manual analysis of the images has shown that the information they contain offers great potential for learning more about the ecology of an introduced species in its new range.

## Graphical abstract

This study uses citizen‐scientist photographic records to examine pollinator‐plant interactions of the wasp *Isodontia mexicana* in its native and introduced range. We use a data sharing method by identifying the plants on iNaturalist images of the wasp using the Pl@ntNet API, a plant species identification application. We validated this method by simultaneously identifying the plants by experts and summarizing other interactions seen in the photographs to support the added value of the image data contributed by the public for ecological research.

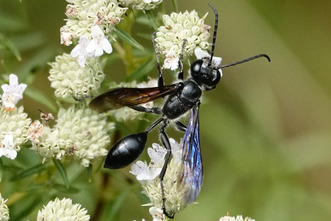

## 1 INTRODUCTION

Biological invasions are a major cause of biodiversity loss, but can also have negative impacts on the economy and human wellbeing (IPBES, 2019). Environmental impacts of alien species are manifested through a variety of mechanisms, but most refer to direct interactions of the invading species with other species, such as predation, herbivory and parasitism (Blackburn et al., 2014; IUCN, 2020). By the time negative impacts of an introduced species are noticed, the species is often too well established to eradicate it, or even manage it effectively (Simberloff et al., 2013). It is obviously important to detect an invasion as early as possible, and to gather information on the impact of a potentially invasive species in the area of interest, so that management options can be effectively prioritized.

We, therefore, need to increase the spatial and temporal coverage of data on biotic interactions as well as the speed of the data collection process, while maintaining a high quality of the data. One of the solutions is to expand data collection by involving the public (Pocock et al., 2018), as has increasingly been done in the last decade in the context of citizen science (Callaghan et al., 2019; Feldman et al., 2021; Pocock et al., 2017). People upload their nature observations supported by multimedia evidence, such as images, on citizen science platforms for biodiversity inventory and monitoring such as iNaturalist, Pl@ntNet and observation.org. These species records contribute to a large proportion of open biodiversity data, about 50% of the data hosted by the Global Biodiversity Information Facility in 2019 (gbif.org, Waller, 2019).

Now judged to be “mainstream” (Callaghan et al., 2019), citizen science has proved its value to track, surveille and manage biological invasions in different forms (Encarnação et al., 2021). For example, various programs are in operation as early warning systems, e.g., for the spread of invasive mosquito species or plant diseases (Brown et al., 2017; Palmer et al., 2017). Moreover, to build support for management, citizen science is also useful, e.g., when the public helps with the elimination or control of invasive species (Miralles et al., 2016). But beside detection and management, citizen science still holds further untapped potential for invasion biology and ecology in general. Specifically, when it comes to monitoring biotic interactions of an introduced species in its new range, the multimedia voucher of the species record (i.e., image, sound, video, text) can be of additional value.

Researchers are not only using the *primary data* (i.e., the observed species together with its observation date and location), but the additional information captured with the citizen observations—the so‐called *secondary data* (Callaghan et al., 2021). Secondary data comprise the information that can be extracted from the observation evidence itself, as a by‐product of the record of a species at a particular time and place. The additional information may include details of the observed species' ecological interactions, morphology, behavior, habitat and various other aspects of its traits and ecology (Pernat et al., 2024).

In this paper, we focus on the secondary data that can be extracted from iNaturalist observations using two different approaches (manually and applying an automated workflow), particularly aiming to increase knowledge on biotic interactions. We selected the Mexican grass‐carrying wasp, *Isodontia mexicana* (Saussure, 1867), Sphecidae, as a case study, and compared its flower preferences in its native and introduced ranges. Such exercises are useful in the assessment of the potential impact of an alien species in an introduced area. The two‐centimeter‐long wasp, nests in small cavities, including bamboo or reed, whose several breeding chambers are lined with plant material and closed with a tuft of grass, the blades of which the female brings one at a time—hence the vernacular name. With North America as its native range, the wasp was introduced in Europe in the middle of the last century with first observations in the South of France (Kelner‐Pillaut, 1962). Since then it has been spreading successfully across Europe (Schirmel et al., 2020) and has been detected in at least 16 European countries so far (Bosch et al., 2018; Burton et al., 2019).

**FIGURE 1:**
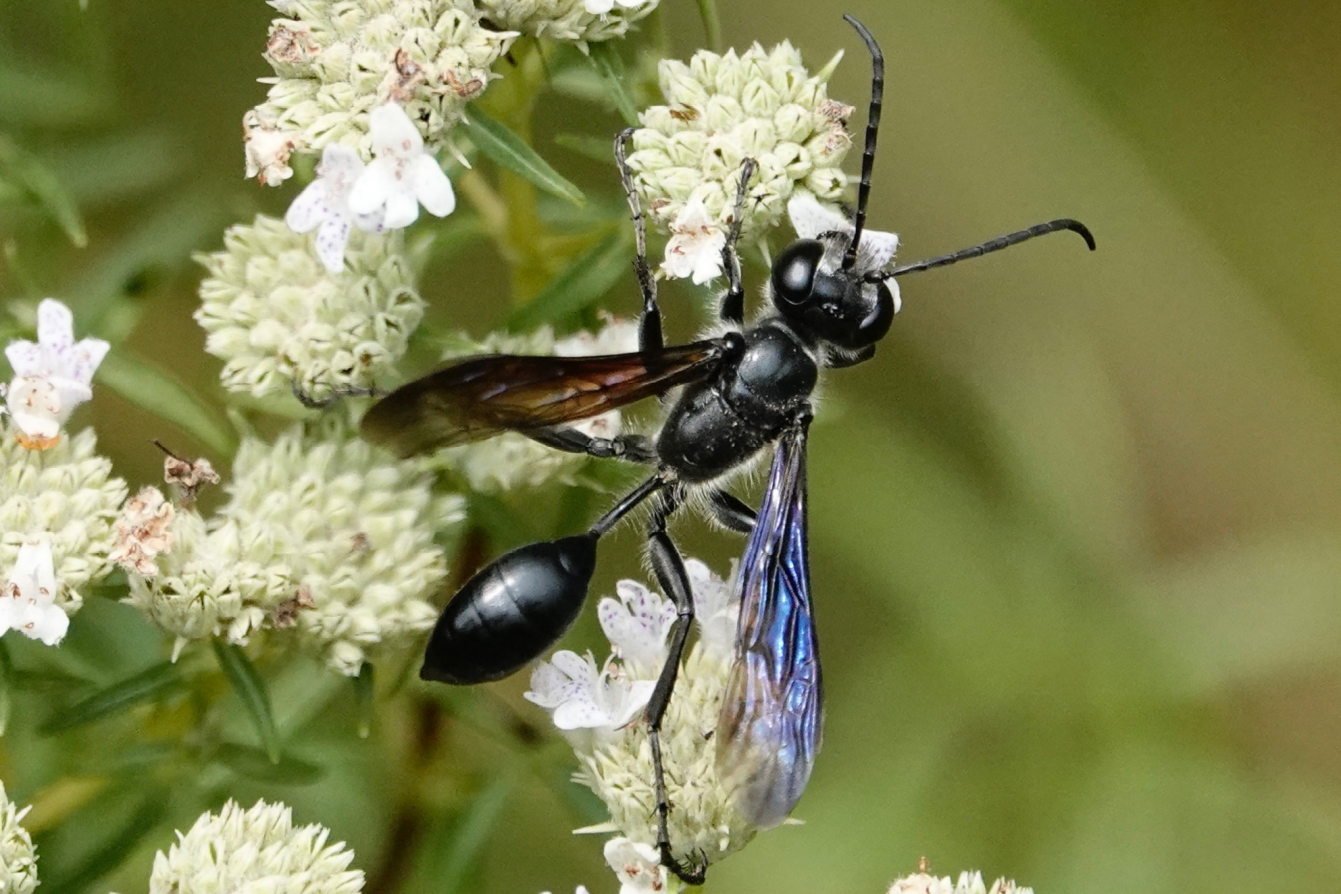
A photograph of *Isodontia mexicana* visiting flowers of *Pycnanthemum tenuifolium* posted to iNaturalist from its native range in North America. https://www.inaturalist.org/observations/91809468, © Louise Woodrich, http://creativecommons.org/licenses/by/4.0/.

This wasp is ideal to investigate plant‐insect interactions for several reasons. First, it has a characteristic and striking appearance and feeds on nectar, which is why it is frequently recorded and mostly on plants (Figure 1). Second, little is known about its potential ecological impacts in Europe (Turrisi, 2020). Third, iNaturalist observations include records from its introduced and native range, which makes a comparison between both ranges possible. Fourth, because of the wasps' behavior of carrying grass leaves and prey for their larvae (i.e., bush‐crickets and crickets) into their nests, other types of interactions may be recorded by citizen scientists in addition to plant visitations that can be extracted as secondary data.

In this study, we investigated the possibility of inferring information about interactions between plants and *I. mexicana* from iNaturalist images. iNaturalist was launched in 2008 by the California Academy of Sciences (iNaturalist, 2022) and has become an independent non‐profit organization for recording and sharing nature's observations worldwide, mostly evidenced through photographs of the organism. It is probably the single largest source of open insect observations globally. These observations are primarily used to identify the species, date, and place, and thus provide scientists with research quality data to understand and protect biodiversity. In contrast to other studies exploiting secondary data, we not only used manually extracted information on plants and interactions from these images (e.g., plant species, flower color and type of interaction), but also applied a novel data exchange approach.

We evaluated the applicability of identifying the host plants of *I. mexicana* by means of the Pl@ntNet API, meaning, we tested whether it is possible to let Pl@ntNet identify the plants on the iNaturalist *I. mexicana* image records. Pl@ntNet, akin to iNaturalist in functionality, focuses on the domain of plant identification using computer vision (Bonnet et al., 2020). Developed in France in 2009, it is one of the pioneering mobile applications exclusively dedicated to discerning plant species (Pl@ntNet, 2022). In this paper we show how iNaturalist images and the Pl@ntNet image classifier can be combined to extract secondary data on flower visitation from citizen science observations. Besides demonstrating the feasibility, our study had the following content‐related and methodological goals

- to assess the accuracy of Pl@ntNet identifications by establishing confidence score thresholds above which identifications agree with botanical experts at species, genus, and family levels.
- to identify possible host plant and flower color preferences of *I. mexicana* in its native and introduced range by means of a secondary data approach and to compare the results with existing literature.
- to identify other biotic interactions captured in the images.

## 2 METHODS

### 2.1 The iNaturalist‐Pl@ntNet automated data exchange workflow

Observations of *I. mexicana* were retrieved from the iNaturalist platform on March 6th, 2022, via the *rinat* package (Barve & Hart, 2021). Only *research grade* observations, those where community consensus has been reached, were used. This resulted in a dataset with 1741 images (see Figure 1 for an example image). Via the image URLs of the iNaturalist pictures, potential plant species displayed on the images as secondary data were identified by Pl@ntNet, applying the command *identify()* from the corresponding *plantnet* package (August, 2019). For two iNaturalist image URLs, Pl@ntNet returned *Species not found* as results, so that the final dataset comprised 1739 images.

We added the columns of suggested species families and genera to the dataset by applying the packages *stringr* (Wickham, 2022) and the command *plantminer()* from the *taxize* package (Chamberlain & Szocs, 2013). Automatically, Pl@ntNet provides a list of plant species suggestions with a corresponding confidence score from 0 to 1 for each candidate that indicates their relative likelihoods. The higher the confidence score, the greater the likelihood that the PlantNet classification model has predicted the correct plant species.

### 2.2 Manual plant identification and validation

To verify the plant species candidates suggested by Pl@ntNet, botanical experts (DM and QG) examined the iNaturalist images of *I. mexicana*, identified plant families and, when possible, genera and species. In some cases, experts categorized plants in images as *unidentifiable* (e.g., insufficient discernable traits for identification) or, if no flower or plant was visible at all, as *noplant*. In addition to the dataset generated by the automated workflow, the color of the flower and the type of interaction was noted. The color of the flower was extracted by only one author (DM) using self‐selected color categories to avoid inconsistencies in rating. In addition to flower color, biotic interactions other than the pollinator‐plant relationship may be randomly detected in the images. Images were visually inspected for other biotic interactions and when discovered, also assigned a category by DM. Besides the wasp feeding on nectar or visiting a flower (both categorized as *visiting*), the insect was photographed with prey (*preying*), resting (*resting*), interacting with humans (e.g., sitting on fingers, *human*), or in other interactions such as mating, dead (also as museum collections) or preyed upon (*other*).

The identifications of plant species from the iNaturalists images were done blind, meaning, the Pl@ntNet suggestions were not provided to the experts, in order to avoid bias. Of the 1739 images, the botanists identified 36.3% plants at species level, 60.3% at genus level and 69.2% at family level (Table 1). No plant was visible on 194 images, so no determination was possible.

**TABLE 1:** Proportion of identifiable plant species, genera and families from iNaturalist images by experts (unidentifiable = plant/flower features not sufficient for identification or beyond the geographical knowledge of the experts).

	Plant species		Plant genus		Plant family	
	*N*	%	*N*	%	*N*	%
Identified	630	36.3	1049	60.3	1204	69.2
Unidentifiable	915	52.6	496	28.5	341	19.6

### 2.3 Statistical analysis

**FIGURE 2:**
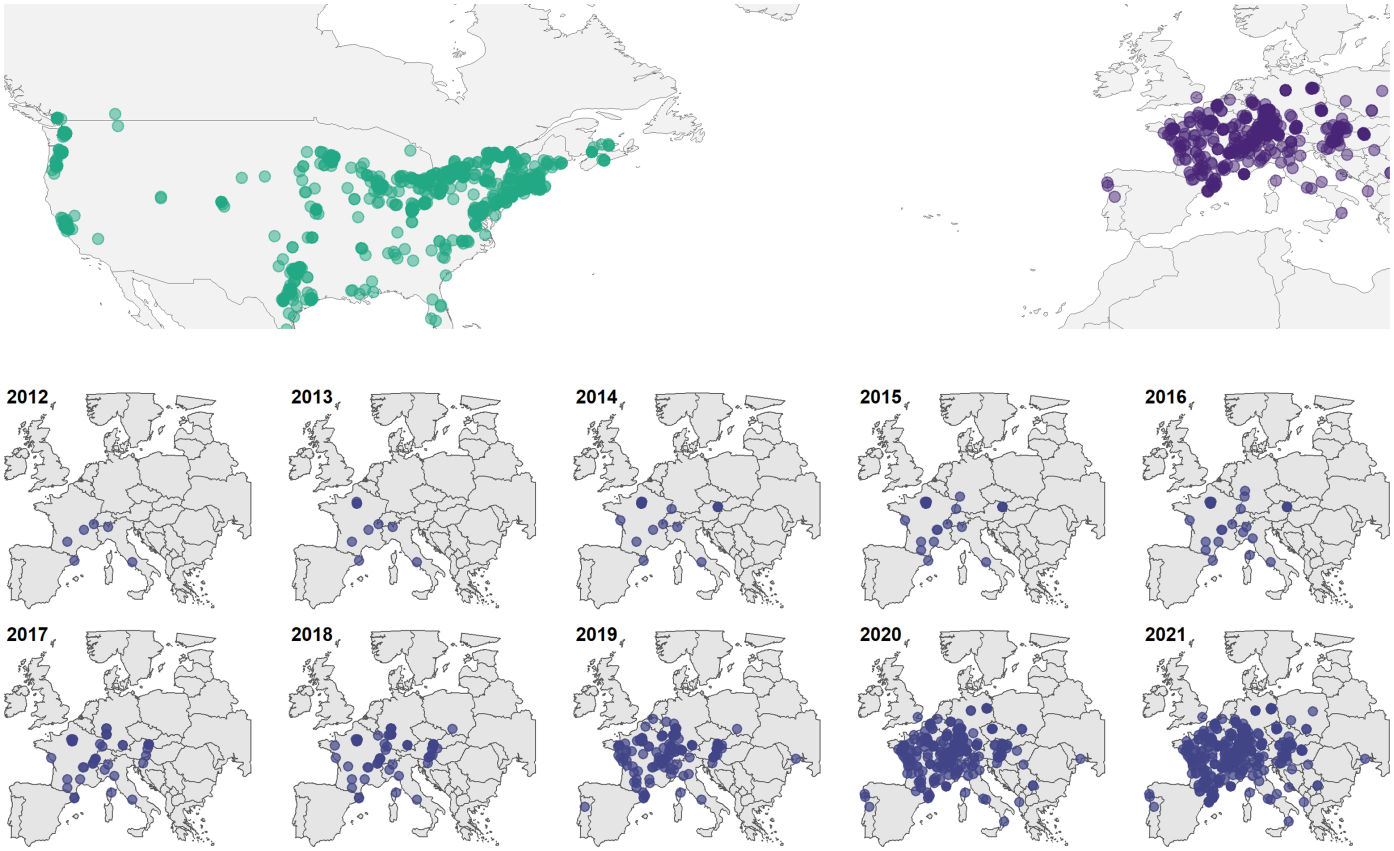
Occurrence of research grade iNaturalist observations of *Isodontia mexicana* used for analysis, cumulative of North America and Europe (top) and over the years, visualizing its apparent spread as documented by citizen scientists in Europe (bottom).

For comparison, the expert dataset was merged with the highest scoring species candidate suggested by Pl@ntNet for each image, so that the comparison was made between the expert identification as benchmark and the most probable (highest scored) plant species suggested by Pl@ntNet. Descriptive analysis, chi‐square and figures were created with R‐4.1.0 (R Core Team, 2022), applying package *tidyverse* (Wickham et al., 2019), *treemapify* (Wilkins, 2021), and *viridis* (Garnier et al., 2021). Range labels—native North America and introduced Europe—were assigned by means of the packages *sf* (Pebesma, 2018) and *rworldmap* (South, 2011). Two observations were lacking geo‐references, around 100 observations needed to be assigned to a continent manually, and two observations were excluded after visual inspection confirmed they originated from Hawaii, and Trinidad and Tobago, respectively. The exclusion was based on the focus of the study on continental North America and the differences in flora and invasion processes compared to islands. As a result, the spatial analysis was conducted with 1735 observations, 577 from Europe and 1158 from North America (Figure 2). Significant differences in frequencies of colors of host flowers were investigated with a Chi‐square test.

## 3 RESULTS

### 3.1 Accuracy of species identification by Pl@ntNet

To obtain a better assessment of Pl@ntNet's performance and accuracy thresholds, we compared the results from the expert identification with the first suggestion (highest score) for each image (mean_score_ = 0.21, median_score_ = 0.15). Table 2 shows the number of images and percentage of matches between Pl@ntNet and the expert identification for different confidence score thresholds, considering only the observations that were identified by the experts (i.e., did not fall into either the *unidentifiable* or the *noplan*t category). The proportion of matches, that is, the agreement in plant genera and families of expert and app identifications, was over 90% from a confidence score of 0.5, while at the species level only 75% of the identifications matched at a score higher than 0.8 (Table 2).

**TABLE 2:** Total and percentage of agreement between Pl@ntNet and expert species identification at different confidence score thresholds up‐ and downward. The higher the score, the more likely it is that the model has predicted the correct plant (species). Therefore, with a confidence score of 0.8, the PlantNet identification is much more likely to be accurate than with a score of 0.5. The observations (*n*) are listed as total observations/observations with expert identification for species, genus and family level. Each percentage value relates to the total observations identified by experts for every level.

Agreement	Plant species		Plant genus		Plant family	
	Cases	% (of *n*_exp_)	Cases	% (of *n*_exp_)	Cases	% (of *n*_exp_)
Score > 0.8 *n*_total_ = 36 *n*_exp_ = 28 | 30 | 34	21	75.0	29	96.7	32	94.1
Score > 0.5 *n*_total_ = 154 *n*_exp_ = 103 | 134 | 146	72	69.9	121	90.3	135	92.5
Score > 0.3 *n*_total_ = 399 *n*_exp_ = 249 | 339 | 371	121	48.6	288	85.0	335	90.3
Score < 0.3 *n*_total_ = 1340 *n*_exp_ = 381 | 710 | 833	88	23.1	393	55.4	613	73.6
All scores *n*_total_ = 1739 *n*_exp_ = 630 | 1049 | 1204	209	33.2	681	64.9	948	78.7

### 3.2 Host plants in the native and introduced ranges

**FIGURE 3:**
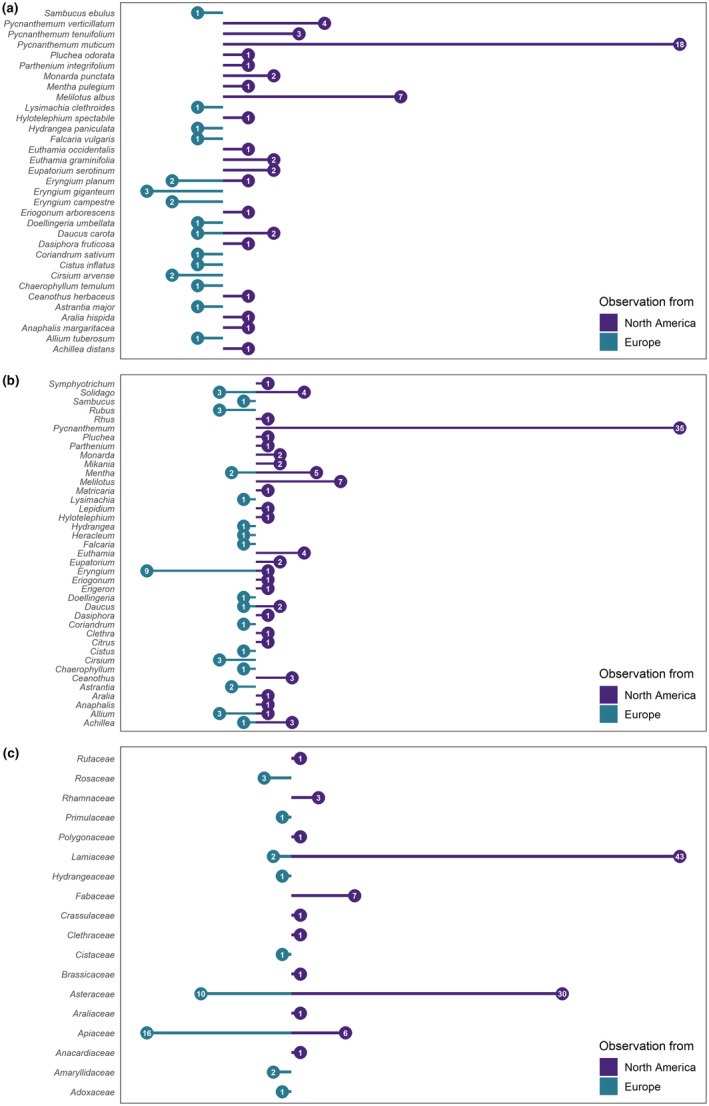
Matching plant species (a), genera (b) and families (c) from expert and Pl@ntNet identification, with scores higher than 0.5.

Figure 3 illustrates for which plant species, genera and families the identification matched between expert and Pl@ntNet's first candidate suggestion with a score higher than 0.5, grouped by native (North America) and introduced range (Europe). To update the plant species list *I. mexicana* interacts with in its introduced region, we tabled the results from the experts verifying the iNaturalist images manually and indicated for which species there was an agreement with Pl@ntNet (Appendix).

### 3.3 Flower color and other types of interactions recorded

**FIGURE 4:**
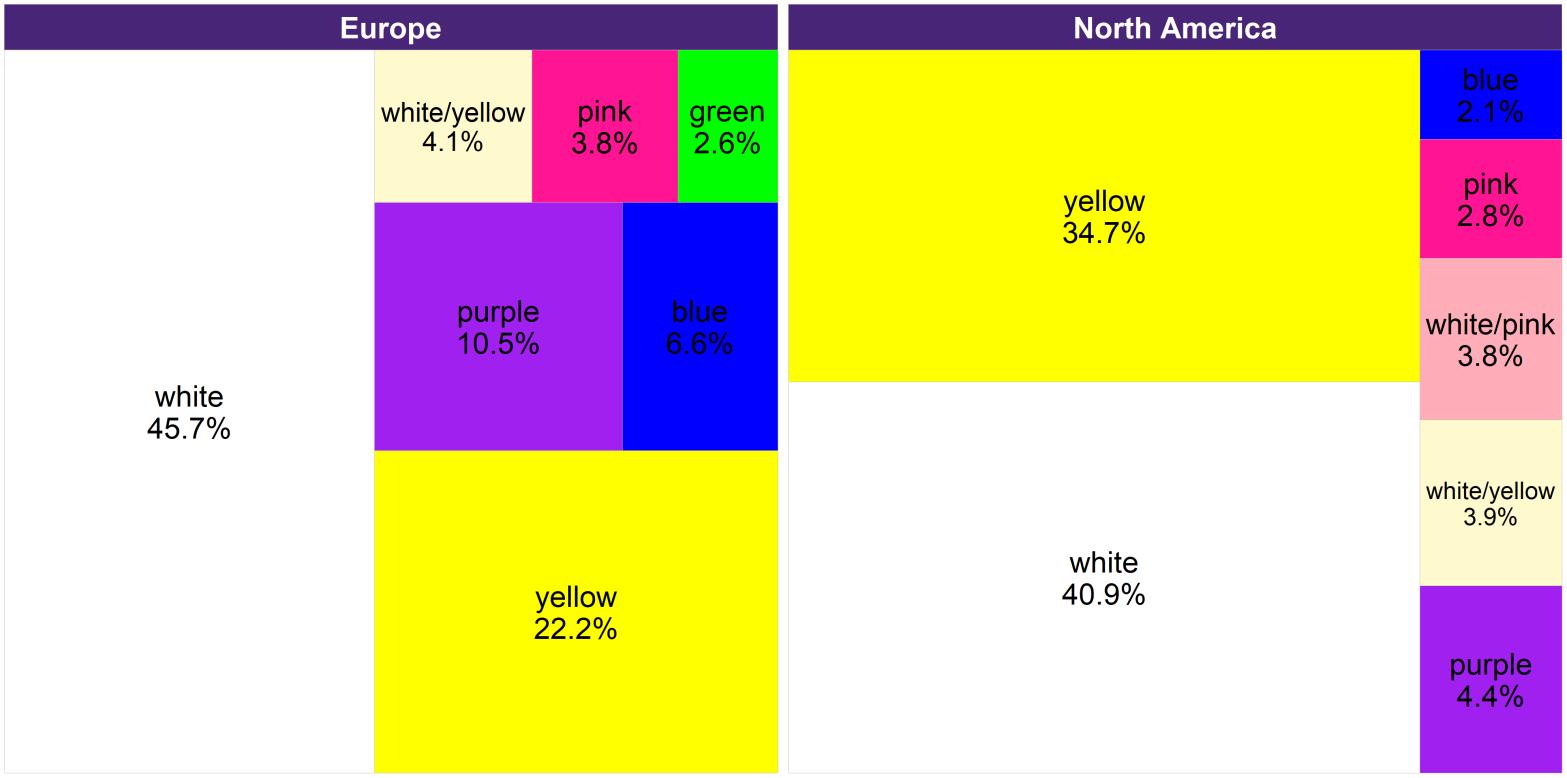
Flower color by continent as classified from iNaturalist images by experts.

The most frequently photographed flower color associated with *I. mexicana* was white, followed by yellow, and then, by a wide margin, purple, blue, and pink. On both continents white accounted for more than 40% of flower colors but the frequency of colors was significantly different (*χ* ^2^ = 69.996, df = 24, *p* < .001). Variability in other hues was greater in Europe than in North America, where yellow accounted for another third of flowers (Figure 4).

**FIGURE 5:**
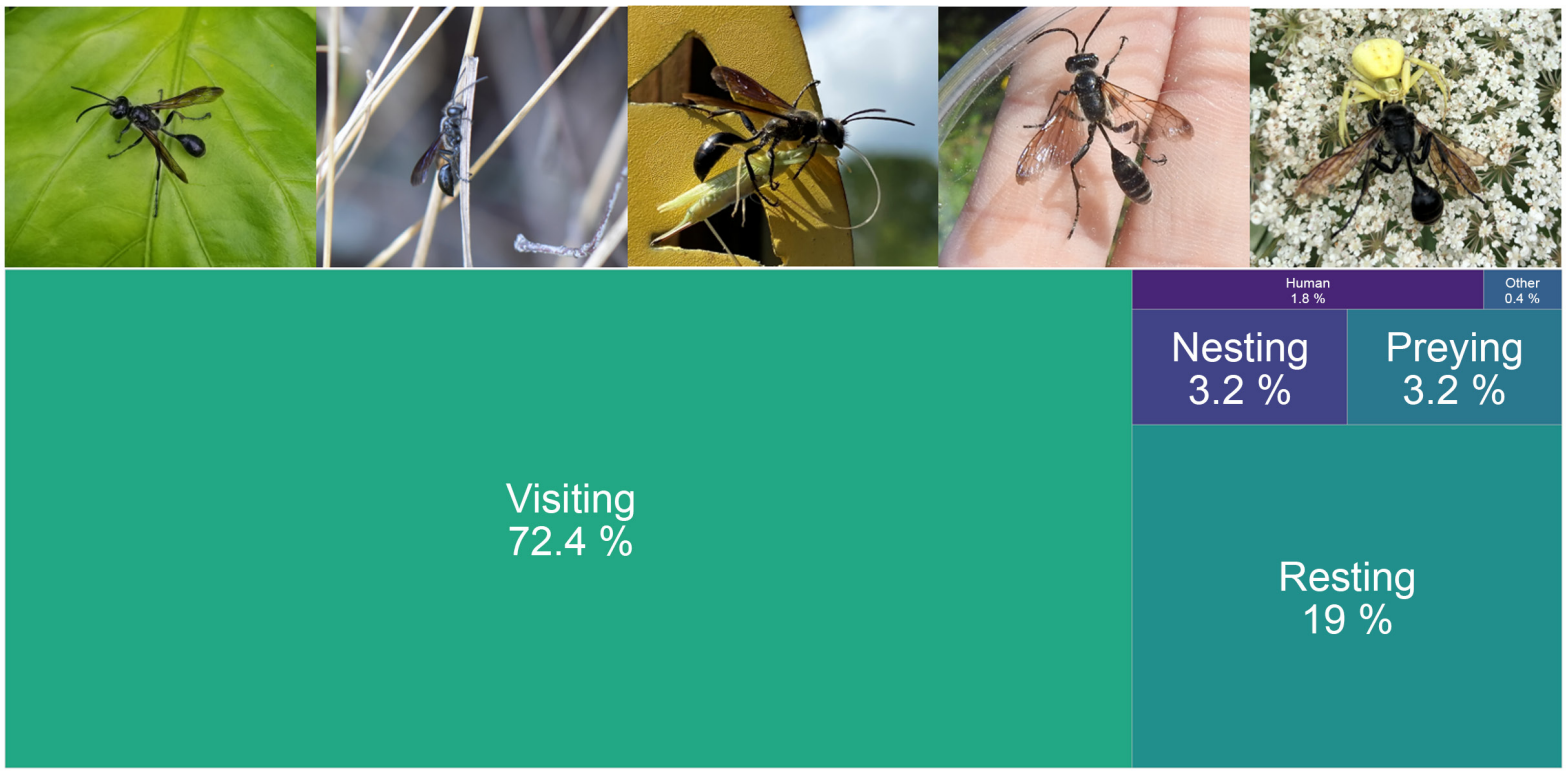
Proportion of different interaction types with example images, from left to right: resting, nesting, catching prey, human interaction and other (here: being preyed upon). The other category also includes images of museum collections, dead, mating or flying wasps. Resting: https://www.inaturalist.org/observations/87310306 © P Raja, https://creativecommons.org/licenses/by‐nc/4.0/; Nesting: https://www.inaturalist.org/observations/91185444 © Jan Becker, https://creativecommons.org/licenses/by‐nc/4.0/; Preying: https://www.inaturalist.org/observations/92995632 © Ryan Leys, https://creativecommons.org/licenses/by‐nc/4.0/; Human: https://www.inaturalist.org/observations/34989495 © Sam Kieschnick, https://creativecommons.org/licenses/by/4.0/; Other: https://www.inaturalist.org/observations/69695395 © haileyeverhart, https://creativecommons.org/licenses/by‐nc/4.0/.

Feeding or sitting on flowers (*visiting*) was also the most common interaction in which the wasp was photographed, but resting on non‐flowering plants was also frequently recorded (Figure 5).

## 4 DISCUSSION

*Isodontia mexicana* is a large and conspicuous insect with a fascinating behavior and is, therefore, frequently recorded on iNaturalist. The wasp is usually photographed sitting on plants and it depends on the photo taking behavior of citizen scientists, which parts of the plant are visible and if these parts suffice for identification. In fact, the plantnet R package had to be updated for our study to prevent Pl@ntNet from rejecting images (about 25%) on which the insect appears too prominently. Rejecting images is a safety feature of Pl@ntNet to avoid images that might violate the privacy of participants (personal communication). For macro images and in many other cases (e.g., plants with no flower, no plant at all), Pl@ntNet provided long lists of suggestions with relatively low scores, and we assume that any agreement of species identification with the experts' assessment at scores lower than 0.3 was likely a coincidence.

However, by reducing the dataset to the first suggestions of Pl@ntNet (candidates with the highest score), we were able to estimate thresholds for the accuracy of Pl@ntNet identifications using the expert identifications as benchmark. For our case study of obtaining taxonomic rank information on pollinator‐plant interactions, the threshold for a 75% correct plant species identification lies above a confidence score of 0.8 and for a more than 90% correct genus and family identification above a confidences score of 0.5. However, only a small number of the images reached values above 0.8 and 0.5 (2.1% and 8.9% of the analyzed image URLs, respectively), but once the programming was done the whole procedure only took a couple of minutes. The experts identified over a third of the plants in the photos at species level (36.6% of the total analyzed image URLs), but the process is more time‐consuming. In addition, more than half of the images could not be used for accurate identification of the plants because they did not display enough distinct plant features for the experts to identify at species level with high confidence.

Consequently, the automatic approach is worthwhile for the explorative analysis of a large number of images within hours, that, for example, experts are capable of manually processing only with a considerable investment of time. Moreover, the use of Pl@ntNet can be practical when working on a broad geographic scale, such as the continents, as even the best botanists do not necessarily have identification skills that span the globe. The results show that determining species level interactions between plants and pollinators using iNaturalist images is challenging for both computers and humans. Therefore, it would be best if this information was provided by the iNaturalist user along with the observation or during community consensus identification. iNaturalist already provides several ways to note interactions in observations, which have already been included in research (Kirchhoff et al., 2021; Maritz & Maritz, 2020). However, we recommend this feature is improved with the use of standard controlled vocabularies and clear guidance on how these fields should be filled in or used.

At the plant genus and family level, Pl@ntNet proved to be reliable even at lower confidence scores. This opens up the possibility of quickly obtaining an overview of host plant richness and frequencies and thus making initial statements about potential insect preferences at these levels. In this way, ecological insights can be gained not only into insects for which little is known concerning their pollination behavior (e.g., mosquitoes and other Diptera species) or that have been recently introduced and for which data on their interactions in their introduced range are lacking. Also, studies that already use iNaturalist occurrence data to model habitat suitability for invasive and native species (Beninde et al., 2023; Dart et al., 2022; Serniak et al., 2023) could refine their models and their predictive power by including secondary data information such as preferred host plant communities and other biotic interactions (Cosentino et al., 2023). For example, Halsch et al. (2020) showed that besides abiotic factors, like minimum winter temperature, the availability of the ornamental host plant, *Passiflora ssp*., in its introduced region of the USA limits the distribution of the gulf fritillary butterfly (*Agraulis vanillae*).

Looking at the species on which the experts and App agree, the plants most frequently visited by *I. mexicana* in its native range, according to iNaturalist observations, are species of the genus *Pycnanthemum*, particularly *Pycnanthemum tenuifolium*, the narrow‐leaved mountain mint. This genus is known to provide rich nectar sources for pollinators (Mader, 2011). It is native to North America and, therefore, does not appear on citizen science images taken in Europe. In Europe, for the genus *Eryngium*, the species *Eryngium giganteum*, *Eryngium planum*, and *Eryngium campestre* are the most common matches of expert and App, as is also the case for *Cirsium arvense*. Of the *Eryngium* species, *Eryngium giganteum* is not native to most European countries, but is native to the steppes of the Middle East and is often planted in parks and gardens as an ornamental. *Eryngium* attracts a wide range of insects (von der Dunk, 2021) and represents the most species‐rich genus in the Apiaceae family. Although some *Eryngium* species are native to North America, there were matches only for the introduced *Eryngium planum*. These results may be the first indication of how a missing plant genus in an introduced range, here *Pycnanthemum* in North America, is replaced in Europe as a source for nectar by a native genus, namely *Eryngium*.

Based on our dataset, the plant families also appear to show very clear preferences of *I. mexicana*, with Lamiaceae being the most often visited in North America due to the frequency of *Pycnanthemum*. However, Asteraceae, Fabaceae and Apiaceae are also commonly visited in the New World, while in Europe only Asteraceae and Apiaceae show higher frequencies. Interestingly, all families except Clethraceae and Araliaceae are distributed worldwide, although according to our analysis most families are visited either in North America or in Europe. Here the limits of interpretation become obvious: the data are probably biased by the absolute abundances of the species of the different plant families, their respective species richness and also probably by their occurrence in proximity to humans, for example, in parks, gardens or recreational areas, or recorder taxonomic preferences (Boakes et al., 2016; Pernat et al., 2021). Therefore, as with the use of citizen science data in other contexts, one has to be careful about interpreting frequencies and proportions. However, this also clearly shows how quickly and on what large scales descriptive information about biotic interactions can be obtained via our data exchange approach that cannot even be collected through decades of fieldwork (see Appendix). The results of plant‐pollinator interactions are largely consistent with what is already known from the literature, but other plants have been found, which *I. mexicana* feeds on or visits, such as *Smyrnium perfoliatum*, *Falcaria vulgaris* or *Euphorbia glareosa*.

The distribution of flower color derived from iNaturalist images shows that *I. mexicana* predominantly visits white and yellow flowers in both North America and Europe. The significant difference in flower color may be due to higher variability in Europe, where purple and blue flowers also reach proportions greater than 5%. Looking only at the frequencies of plant families on which App and experts agreed, one might infer from field experience that Apiaceae and Asteraceae frequently have white or yellow flowers, while Lamiaceae species frequently flower white, or purple and pink. But studies looking at the frequency of flower colors in these families and possible geographic differences seem to be lacking on this large spatial scale to confirm the anecdotal impression. Our approach shows the feasibility and potential to retrieve flower color information from citizen science data. To our knowledge, only the study of Catron et al. (2023) utilized citizen science data to learn about color preferences of two soldier beetle species across the USA. However, it is slow to do manually, and with thousands of images, there is a need for a method to automatically identify flower color from the images. Recent publications using, for example, k*‐means clustering*, to extract flower colors, give hope for automation in the near future (Gibert et al., 2022; Perez‐Udell et al., 2023).

Similarly promising to extracting flower coloration—and similarly time‐consuming if done manually—is classifying and analyzing other biotic interactions captured in the images by iNaturalist observers. However, this study indicated that the PlantNet classifier would be suitable for filtering out images that do not show plants by applying a lower confidence threshold. In this way, images could be pre‐selected and then visually inspected in a hybrid intelligence approach (Rafner et al., 2022). The dataset contained a few but fascinating photos of the wasp, which lives up to its name by carrying or biting off grass leaves and transporting grasshoppers and crickets in its clutches to nests. Images from citizen science projects, as well as from social media, are useful for learning more about, for example, natural enemies or the diet of target species over a larger geographic area (Maritz & Maritz, 2020; Panter & Amar, 2021). For *I. mexicana*, preyed bush‐cricket and cricket species have only been surveyed in country‐specific field studies (Amiet, 2009; Bitsch, 2010; Scaramozzino & Currado, 1988; Tussac & Voisin, 1989; Westrich, 1998) where, for example, in Germany the entry of species into artificial nesting aids was evaluated (Schirmel et al., 2020). Here, despite the small proportion of 52 images (3.2%), a closer look at the preyed orthopterans or other species would be worthwhile to expand or confirm the state of knowledge.

In addition to the above‐mentioned caveats regarding the accuracy of identification and the restricted number of interpretable images, there are some other specific and general limitations of this workflow. Pl@ntNet only allows a limited number of images for identification per day per user, so a comprehensive analysis can only take place in coordination with the providers or over a longer period of time. When interpreting the frequencies of plant species, genera and families visited, it should be noted that the Pl@ntNet model may not be able to identify the different taxonomic groups equally well. This would distort the results, making it difficult to speak of pollinator preferences.

There are some general limitations when working with secondary data from open sources such as citizen science platforms or social media, that also apply here, for example data bias. Which plants the citizen scientists prefer to photograph may be a factor that influences the frequency of plant species as well as the flower colors. Lastly, using secondary data could raise ethical concerns, especially regarding privacy, and consent (Di Minin et al., 2021), which the user needs to be aware of, particularly if the observer becomes the subject of the research.

## 5 CONCLUSIONS

We demonstrate that our approach and Pl@ntNet can be a valuable and efficient tool for genus and family level investigations of insect flower visitations from citizen science images. For high confidence scores, even species identification with Pl@ntNet is worthwhile, given that the results could be reviewed by experts in a non‐blind hybrid intelligence approach, which reduces the workload significantly. Working with low confidence values could allow researchers to automatically pre‐select images that do not feature flowers, but potentially other interactions.

At a time when expert knowledge and availability are scarce, Pl@ntNet cannot replace them in terms of quality, but it can provide solid support in terms of quantity. Especially for ecologists who do not have the skills or resources to develop custom computer vision models, open access to pretrained models allows them to use those in innovative ways. As a method in interaction ecology and, as shown in this case study, particularly in the context of invasion biology, evaluation of secondary data provides valuable information on biotic and abiotic interactions of introduced species to better assess their impacts on native flora and fauna.

We can envision studying the novel resource use of insects in cities with a high proportion of introduced plants, particularly in urban novel ecosystems—even in international approaches. Beyond invasion biology, phenological studies are conceivable, such as the shift of food plants over the activity period of a species. Besides typical pollinators such as butterflies, bees or hoverflies, food plants of herbivorous insects from the Orthoptera or Hemiptera groups could also be investigated. Secondary data can also be used as complementary data for field experiments to supplement them or to validate insights by up‐scaling. Many areas of application are imaginable, but the potential of secondary data must now be utilized and tested by the scientific community.

## AUTHOR CONTRIBUTIONS

**Nadja Pernat:** Conceptualization (lead); formal analysis (lead); investigation (lead); methodology (lead); visualization (lead); writing – original draft (lead); writing – review and editing (lead). **Daniyar Memedemin:** Formal analysis (supporting); investigation (equal); writing – review and editing (supporting). **Tom August:** Software (lead); writing – review and editing (supporting). **Cristina Preda:** Funding acquisition (lead); writing – review and editing (supporting). **Lien Reyserhove:** Investigation (supporting); writing – review and editing (supporting). **Jens Schirmel:** Investigation (supporting); writing – review and editing (supporting). **Quentin Groom:** Formal analysis (supporting); funding acquisition (supporting); investigation (supporting); methodology (supporting); writing – review and editing (equal).

## CONFLICT OF INTEREST STATEMENT

The authors declare no competing interests.

## Data Availability

The R code and corresponding data is available via https://zenodo.org/records/11185002.

## 1

Table of plant species visited *by Isodontia mexicana* based on the expert identifications and grouped by region, also showing the frequency of the identified plants (*n*_identification_) and number of matches with Pl@ntNet not considering any score threshold (*n*_matches_).

**Table:**

Species (by expert identification)	Continent	*n*_identification_	*n*_matches_	Already published in
*Mentha suaveolens*	Europe	28	5	Bosch et al. (2018)
*Eryngium planum*	Europe	24	12	Bosch et al. (2018), Burger (2009), Hausl‐Hofstätter & Teppner (2015), Rennwald (2005), Saure et al. (2019), Tischendorf (2016)
*Eryngium campestre*	Europe	22	6	Bosch et al. (2018), Burger (2015)^a^, Diéguez Fernández (2019), Hamon et al. (1988)^a^, Tischendorf (2016), Westrich (2007)
*Foeniculum vulgare*	Europe	16	6	Hamon et al. (1988)^a^
*Solidago canadensis*	Europe	14	0	Amiet (1989)^a^, Bengus (2022), Bosch et al. (2018), Gradinarov (2017)^a^, Hopfenmüller (2016)^a^, Rennwald (2005), Saure et al. (2019), Tischendorf (2016), Vernier (1995)^a^, Weiser (2018)^a^
*Achillea millefolium*	Europe	10	1	Bosch et al. (2018), Djamal (2012)^a^, Vernier (1995), Westrich (1998)
*Mentha spicata*	Europe	9	2	Bosch et al. (2018)
*Daucus carota*	Europe	9	4	Bosch et al. (2018), Tischendorf (2016)
*Hedera helix*	Europe	7	0	Saure et al. (2019)
*Melilotus albus*	Europe	6	5	Hiermann (2020), Vernier (1995), Hamon et al. (1988)^a^
*Erigeron annuus*	Europe	6	1	Bengus (2022), Vernier (1995), Weiser (2017)^a^
*Eryngium giganteum*	Europe	5	5	
*Cirsium arvense*	Europe	5	3	Bosch et al. (2018)
*Smyrnium perfoliatum*	Europe	4	0	
*Sambucus ebulus*	Europe	4	2	Diéguez Fernández (2019)
*Mentha longifolia*	Europe	4	1	Bosch et al. (2018), Cétkovic et al. (2012)
*Solidago gigantea*	Europe	3	0	Bosch et al. (2018), Rennwald (2005), Weiser (2017)^a^
*Falcaria vulgaris*	Europe	3	2	
*Euphorbia glareosa*	Europe	3	0	
*Solidago virgaurea*	Europe	2	0	Bosch et al. (2018), Rennwald (2005), Tischendorf (2016)
*Seseli annuum*	Europe	2	1	
*Ruta graveolens*	Europe	2	2	
*Origanum vulgare*	Europe	2	2	
*Lysimachia clethroides*	Europe	2	1	
*Knautia drymeia*	Europe	2	0	
*Hydrangea paniculata*	Europe	2	2	
*Heracleum sphondylium*	Europe	2	0	
*Euthamia graminifolia*	Europe	2	2	
*Urtica dioica*	Europe	1	0	
*Tripleurospermum inodorum*	Europe	1	0	
*Torilis arvensis*	Europe	1	0	
*Symphyotrichum pilosum*	Europe	1	1	
*Symphiotrychum lanceolatum*	Europe	1	0	
*Spiraea japonica*	Europe	1	1	
*Rubus caesius*	Europe	1	0	
*Plantago lanceolata*	Europe	1	1	
*Pastinaca sativa*	Europe	1	0	Hamon et al. (1988)^a^
*Orlaya grandiflora*	Europe	1	1	
*Mentha pulegium*	Europe	1	1	Bosch et al. (2018)
*Mentha arvensis*	Europe	1	1	Bosch et al. (2018)
*Lobularia maritima*	Europe	1	1	
*Levisticum officinale*	Europe	1	0	Kiefer (2018)^a^
*Hypericum perforatum*	Europe	1	0	
*Hylotelephium telephium*	Europe	1	1	
*Hylotelephium spectabile*	Europe	1	0	
*Hydrangea heteromalla*	Europe	1	0	
*Gypsophila paniculata*	Europe	1	1	Weiser (2017)^a^
*Eupatorium cannabinum*	Europe	1	1	
*Eryngium aquaticum*	Europe	1	0	
*Erica vagans*	Europe	1	0	
*Echium italicum*	Europe	1	0	
*Doellingeria umbellata*	Europe	1	1	
*Coriandrum sativum*	Europe	1	1	
*Cistus inflatus*	Europe	1	1	
*Chaerophyllum temulum*	Europe	1	1	
*Campsis radicans*	Europe	1	0	
*Berteroa incana*	Europe	1	1	
*Astrantia minor*	Europe	1	0	
*Astrantia major*	Europe	1	1	
*Allium tuberosum*	Europe	1	1	
*Agastache scrophulariifolia*	Europe	1	0	
*Aesculus parviflora*	Europe	1	1	
*Aegopodium podagraria*	Europe	1	0	Schmidt (2015)^a^
*Achillea setacea*	Europe	1	0	
*Solidago canadensis*	North America	61	2	
*Pycnanthemum muticum*	North America	46	31	
*Mentha suaveolens*	North America	24	1	
*Daucus carota*	North America	23	14	
*Pycnanthemum tenuifolium*	North America	19	6	
*Foeniculum vulgare*	North America	15	3	
*Melilotus albus*	North America	12	10	
*Eryngium planum*	North America	12	6	
*Solidago gigantea*	North America	10	1	
*Mentha spicata*	North America	10	1	
*Eupatorium perfoliatum*	North America	7	3	
*Anaphalis margaritacea*	North America	7	2	
*Achillea millefolium*	North America	7	0	
*Pycnanthemum verticillatum*	North America	6	4	
*Hedera helix*	North America	6	0	
*Pycnanthemum incanum*	North America	5	1	
*Euthamia graminifolia*	North America	5	3	
*Lobularia maritima*	North America	4	3	
*Ilex aquifolium*	North America	4	0	
*Hydrangea paniculata*	North America	4	1	Voith and Seidler (2015)^a,b^
*Eriogonum elatum*	North America	4	0	
*Coriandrum sativum*	North America	4	3	Dardaine and Péru (2011)^a,b^
*Cirsium arvense*	North America	4	1	
*Campsis radicans*	North America	4	0	
*Achillea distans*	North America	4	1	
*Monarda punctata*	North America	3	2	
*Mentha longifolia*	North America	3	0	
*Eupatorium serotinum*	North America	3	2	
*Eryngium yuccifolium*	North America	3	1	
*Erigeron annuus*	North America	3	1	
*Apocynum cannabinum*	North America	3	1	
*Symphyotrichum lanceolatum*	North America	2	0	
*Solidago virgaurea*	North America	2	0	
*Polytaenia nuttallii*	North America	2	1	
*Orlaya grandiflora*	North America	2	0	
*Hylotelephium spectabile*	North America	2	1	
*Hydrangea heteromalla*	North America	2	0	
*Euonymus japonicus*	North America	2	1	
*Asclepias curassavica*	North America	2	0	
*Ampelopsis cordata*	North America	2	0	
*Torilis arvensis*	North America	1	0	
*Tagetes erecta*	North America	1	1	
*Spiraea salicifolia*	North America	1	0	
*Spiraea chamaedryfolia*	North America	1	0	
*Solidago uliginosa*	North America	1	0	
*Solidago petiolaris*	North America	1	1	
*Solida gogigantea*	North America	1	0	
*Smyrnium perfoliatum*	North America	1	0	
*Sium suave*	North America	1	1	
*Sisymbrium officinale*	North America	1	0	
*Sedum urvillei*	North America	1	0	
*Rhus typhina*	North America	1	0	
*Pycnanthemum pilosum*	North America	1	0	
*Polyatenia texana*	North America	1	0	
*Pluchea odorata*	North America	1	1	
*Petroselinum crispum*	North America	1	0	
*Persicaria lapathifolia*	North America	1	0	
*Pastinaca sativa*	North America	1	0	
*Parthenium integrifolium*	North America	1	1	
*Muehlenbeckia complexa*	North America	1	1	
*Monarda fistulosa*	North America	1	1	
*Mikania scandens*	North America	1	0	
*Mentha pulegium*	North America	1	1	
*Levisticum officinale*	North America	1	0	
*Lepidium campestre*	North America	1	0	
*Lamatium columbianum*	North America	1	0	
*Hylotelephium telephium*	North America	1	1	
*Hemerocallis fulva*	North America	1	0	
*Euthamia occidentalis*	North America	1	1	
*Eurybia macrophylla*	North America	1	0	
*Euphorbia esula*	North America	1	0	
*Eupatorium cannabinum*	North America	1	0	
*Eriogonum grande*	North America	1	1	
*Eriogonum giganteum*	North America	1	0	
*Eriogonum arborescens*	North America	1	1	
*Dasiphora fruticosa*	North America	1	1	
*Cornus sanguinea*	North America	1	0	
*Clethra alnifolia*	North America	1	0	
*Clematis virginiana*	North America	1	1	
*Ceanothus herbaceus*	North America	1	1	
*Ceanothus americanus*	North America	1	1	
*Baccharis douglasii*	North America	1	1	
*Astrantia major*	North America	1	0	
*Asclepias syriaca*	North America	1	1	
*Asclepia tuberosa*	North America	1	0	
*Aralia hispida*	North America	1	1	
*Amphiachrys dracunculoides*	North America	1	0	
*Ageratina altissima*	North America	1	0	
*Abies alba*	North America	1	0	

*Note*: The following plants with documented visits from *I. mexicana* were not detected in this study, for Europe: *Budellja spec*. (Vernier, 1995), *Bupleurum rotundifolium* (Hamon et al., 1988 ^a^), *Cenothus* ‘Gloire de Versailles’ (Smit & Wijngaard, 2010), *Echinophora ritro* (Dardenne, 2013 ^a^), *Echinophora spinosa* (Hamon et al., 1988 ^a^), *Eryngium maritimum* (Hamon et al., 1988 ^a^), *Euonymus europaeus* (Hamon et al., 1988 ^a^), *Fallopia japonica* (Hausl‐Hofstätter & Teppner, 2015), *Galatella sedifolia* (Hamon et al., 1988 ^a^), *Lycopus europaeus* (Notton, 2016 ^a^), *Mentha & piperita* (Friebe, 2015; Kiefer, 2018^a^), *Peucedanum oreoselinum* (Weiser, 2017^a^), *Polygonum cuspidatum* (Vernier, 1995), *Reynoutria japonica* (Gradinarov, 2017 ^a^, Hausl‐Hofstätter & Teppner, 2015 ^a^, Vernier, 1995 ^a^), *Sambucus nigra* (Hausl‐Hofstätter & Teppner, 2015), *Tamarix gallica* (Hamon et al., 1988 ^a^) and for North America, *Acer rubrum* (L.) and *Ailanthus altissima* (Elmquist et al., 2023).

^a^These references were extracted from Burton et al. (Table 1, 2019).

^b^These host plants were documented for Europe in the indicated publications.
